# Long-term efficacy and impact on rehospitalization of Qishen granules in patients with hypertension and heart failure with reduced ejection fraction: a multicenter, randomized, double-blind, placebo-controlled trial protocol

**DOI:** 10.3389/fcvm.2026.1897966

**Published:** 2026-09-07

**Authors:** Fuyuan Zhang, Zhaorui Cui, Zirui Wang, Yonghao Li, Tiantian Xue, Ruikang Liu, Jun Li

**Affiliations:** 1 Cardiovascular Department, Guang'anmen Hospital, China Academy of Chinese Medical Sciences, Beijing, China; 2 Postdoctoral Station of China Academy of Chinese Medical Sciences, Beijing, China; 3 Graduate School, Beijing University of Chinese Medicine, Beijing, China

**Keywords:** Chinese medicine, clinical protocol, HFREF, hypertension, RCT - randomized controlled trial

## Abstract

**Background:**

Heart failure with reduced ejection fraction (HFrEF) represents a severe manifestation or terminal stage of various cardiac diseases. Long-term hypertension frequently leads to pathological changes such as ventricular remodeling, resulting in HFrEF. Despite continuous advancements in medications targeting hypertension complicated by HFrEF, the five-year survival rate for patients remains below 50%. Chinese Medicine (CM) demonstrates favorable long-term efficacy in managing HFrEF and reducing readmission rates. Focusing on the primary symptoms of HFrEF, we innovatively proposed the syndrome pattern “Toxic Pathogen Syndrome” and developed the effective compound formula Qi Shen Granules (QSG). This trial aims to conduct a prospective clinical study to evaluate the efficacy and safety of QSG in patients with hypertension and HFrEF.

**Methods:**

This is a multicenter, prospective, randomized, double-blind, placebo-controlled clinical trial. A total of 220 eligible participants with hypertension and HFrEF will be recruited and randomized in a 1:1 ratio to receive either QSG or placebo for 12 weeks. All participants received guideline-directed treatment for hypertension and HFrEF throughout the study period. The primary outcome is the improvement rate in plasma NT-proBNP levels from baseline to endpoint and the six-minute walk test. Secondary outcomes include echocardiographic parameters, NYHA functional class, Kansas City Cardiomyopathy Questionnaire (KCCQ), and Heart Failure Toxicity Scale. Safety measures, including liver and kidney function blood tests and adverse events, were used to assess QSG safety. We also conducted follow-up assessments at weeks 26 and 52 to evaluate patient readmission rates and the occurrence of cardiovascular adverse events.

**Discussion:**

The results of this study may establish clinical evidence supporting the therapeutic benefits and safety of QSG in the management of hypertension with HFrEF.

**Clinical Trial Registration:**

http://itmctr.ccebtcm.org.cn/, ITMCTR2025000665.

## 1 Introduction

Heart failure with reduced ejection fraction (HFrEF) represents a severe manifestation or terminal stage of various cardiac diseases (1). The global prevalence of heart failure continues to rise. Although drug development has improved clinical outcomes, numerous challenges persist: high hospitalization and mortality rates, with a five-year survival rate still below 50%; intractable issues such as myocardial fibrosis and ventricular remodeling remain unresolved; suitable medications remain unavailable for certain populations, such as those with concomitant renal insufficiency; and long-term medication use carries toxic side effects (2–5).

Chinese Medicine (CM) Demonstrates Significant Efficacy in Treating Heart Failure (6). The enhanced efficacy of modern medicine stems from updated treatment concepts and the discovery of new therapeutic targets, while innovations in understanding pathogenesis are the prerequisite and key to breakthroughs in CM treatment. Although numerous Chinese patent medicines and compound formulas exist for treating heart failure, they predominantly focus on therapeutic approaches such as “Yiqi, Wenyang, Huoxue”, with insufficient innovation in pathogenesis and treatment methodology. Through long-term clinical practice, we observed that patients exhibiting restlessness, dry mouth, thirst, dry stools, rapid pulse, and red tongue with yellow coating frequently experienced acute heart failure episodes or rapid disease progression. Incorporating herbs with detoxifying properties into formulations improved these symptoms and yielded superior treatment outcomes. Therefore, we propose that “toxic pathogens” represent a novel pathogenesis in hypertension complicated by HFrEF. This led to the development of Qishen Granules (QSG), an innovative CM characterized by its detoxifying properties and significant therapeutic efficacy.

Multiple animal and cellular studies indicate that QSG modulates inflammatory pathways to inhibit myocardial injury and improve chronic myocardial ischemia (7, 8). Further clinical studies indicate QSG may serve as a safe adjunctive therapeutic strategy for patients with congestive heart failure (9). However, large-scale clinical research targeting hypertension complicated by HFrEF remains insufficient. Therefore, we designed a multicenter, double-blind, randomized, placebo-controlled trial to investigate the efficacy and safety of QSG in patients with hypertension and HFrEF.

## 2 Methods

### 2.1 Drugs

QSG are composed of six Chinese herbal ingredients: Astragalus membranaceus 30 g, Aconitum carmichaeli 9 g, Salvia miltiorrhiza 15 g, Lonicera japonica 10 g, Leonurus japonicus 30 g, and Fried Glycyrrhiza uralensis 6 g (Table 1), manufactured by Jiangyin Tianjiang Pharmaceutical Co., Ltd. Granule preparation follows validated procedures. In brief, the herbal medicines are decocted in water, ethanol precipitated, and the extract concentrated under reduced pressure. It is then vacuum-dried and mixed with lactose as an excipient. The placebo contains 5% active ingredients, 93.9% maltodextrin, 1.0% mixed colorants, and 0.1% bittering agent. The placebo is identical to the test agent in color, specifications, packaging, labeling, and content appearance. The herbal materials used for QSG were supplied with manufacturer-issued certificates documenting their botanical origin, geographic source, processing method, pharmacopoeial basis, and material batch number. For safety control, Aconitum carmichaelii was used exclusively in the processed form of Heishunpian rather than as crude Aconitum, and its processing complied with the Chinese Pharmacopoeia 2020. The two blinded investigational products were manufactured by Jiangyin Tianjiang Pharmaceutical Co., Ltd. under batch No. 2411342 and underwent batch-release testing according to the Chinese Pharmacopoeia 2020 and the manufacturer's internal quality specifications. Sixty sachets of each product were sampled for testing. Both products met the predefined requirements for appearance, complete solubility, fill-weight variation and granularity; their moisture contents were 4.0% and 5.0%, respectively. For both products, the total aerobic microbial count and the total yeast and mould count were below 10 cfu/g, and Escherichia coli was not detected. The batch was therefore released as compliant, and the same source-traceability and batch-release requirements will be applied to all investigational products used during the trial.

**Table 1:** Specific ingredients of QSG.

Herbal name	Latin scientific name	Plant part	Weight ratio (g)
Huangqi	*Astragalus membranaeus* (Fisch.)Bge*.*	Roots	30
Fuzi	*Aconitum carmichaelii* Debx.	Roots	9
Danshen	*Sal*via *miltiorrhiza* Bge.	Roots	15
Jinyinhua	*Lonicera japonica* Thunb.	Roots	10
Yimucao	*Leonurus japonicus* Houtt.	Roots	30
Gancao	* Glycyrrhiza uralensis* Fisch*.*	Roots	6

### 2.2 Design

**Figure 1:**
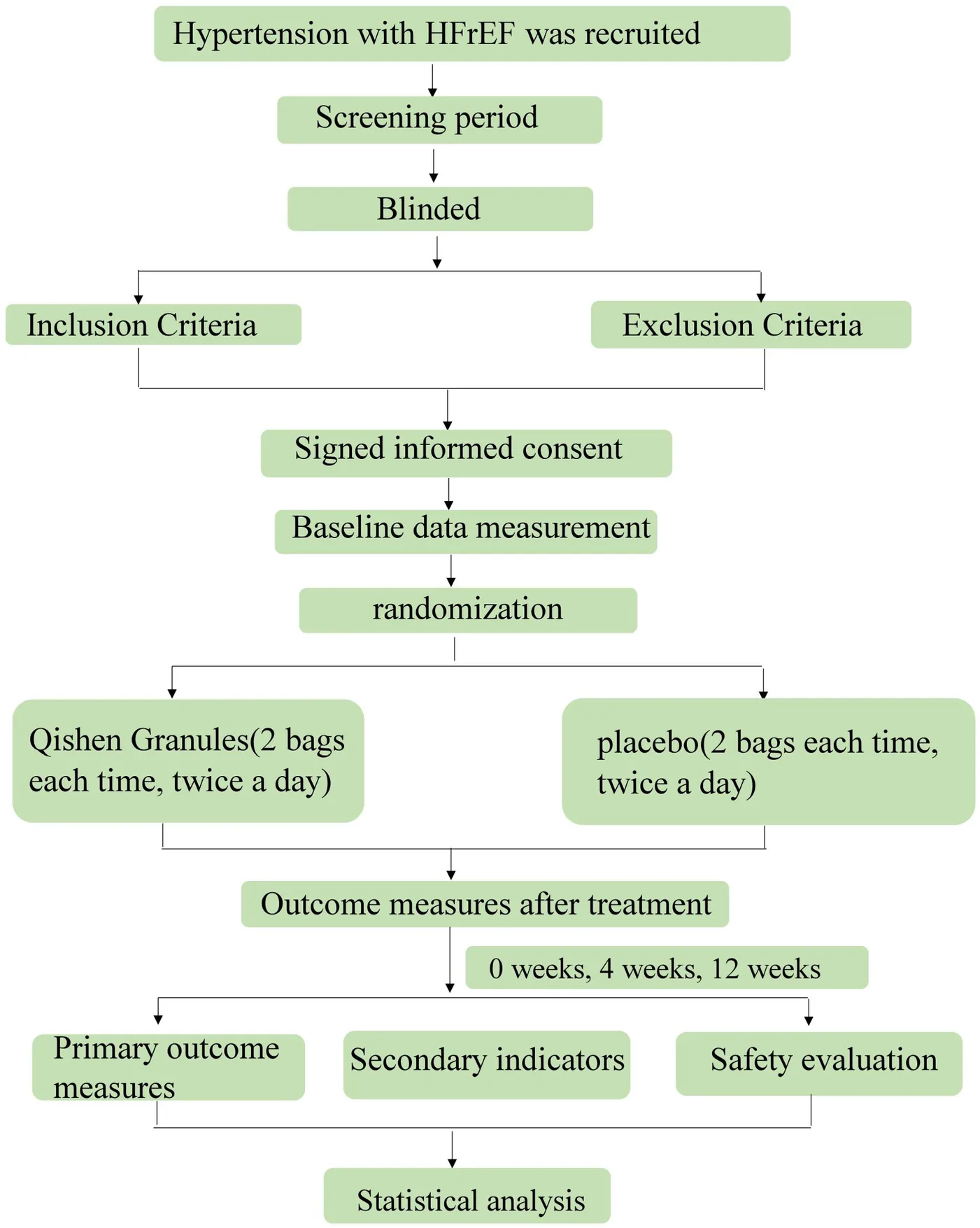
Research flowchart.

This study is a randomized, double-blind, placebo-controlled, parallel-group, multicenter clinical trial supported by the Key Research and Development Program of the Ministry of Science and Technology in China. It was conducted among patients with hypertension complicated by HFrEF. The study was conducted simultaneously at Guang'anmen Hospital of China Academy of Chinese Medical Sciences, Affiliated Hospital of Shandong University of Chinese Medicine, Langfang People's Hospital, Weifang Hospital of Traditional Chinese Medicine, Affiliated Hospital of Xinxiang Medical University, Beijing Changping District Hospital of Traditional Chinese Medicine, Shanxi Provincial Hospital of Traditional Chinese Medicine, China-Japan Friendship Hospital of Jilin University, Linyi Hospital of Traditional Chinese Medicine, Chaoyang Central Hospital, and Yantai Mountain Hospital, enrolling a total of 220 patients. After providing written consent, individuals with hypertension and HFrEF were randomly assigned to receive QSG or placebo for 12 weeks. Standardized treatment followed medication regimens specified in 2017 ACC/AHA/AAPA/ABC/ACPM/AGS/APhA/ASH/ASPC/NMA/PCNA Guideline (10) and the 2022 AHA/ACC/HFSA Heart Failure Management Guidelines (3). Drug types and doses were fixed (unless contraindicated or intolerable). Figure 1 illustrates the study flowchart.

### 2.3 Confirmation of case count

The sample size was calculated based on the expected reduction in serum NT-proBNP levels. Consistent with previous studies demonstrating that a > 30% reduction in NT-proBNP is associated with significantly better prognosis compared to stable or rising levels, efficacy was defined as a > 30% decrease from baseline (11). Based on preliminary research data, the efficacy rates of QSG and placebo after 12 weeks of intervention in chronic heart failure were 50% and 32%, respectively (9). Furthermore, pre-experimental studies demonstrated that QSG significantly improved cardiac function, cardiac structure, and factors related to myocardial hypertrophy in mice with transverse aortic constriction (TAC)-induced heart failure. Therefore, this study projected an efficacy rate of 53% for QSG and 33% for placebo. Given a type-I error rate of *α* = 0.05 and a type-II error rate of *β* = 0.2, and accounting for a 15% dropout rate, the required sample size was calculated using the two-sample comparison of proportions in *PASS* (version 21.0.3) software. This yielded 110 patients per group, totaling 220 cases for both groups.

### 2.4 Diagnostic criteria

#### 2.4.1 Hypertension

Hypertension is defined as clinic blood pressure ≥140/90 mmHg in untreated patients, home blood pressure ≥135/85 mmHg, or 24-hour ambulatory blood pressure ≥130/80 mmHg (daytime ≥135/85 mmHg or nighttime ≥120/70 mmHg) (10).

#### 2.4.2 Heart failure

Patients must have a history of hypertension and dyspnea. Physical examination should reveal pertinent signs, including pulmonary rales, bilateral lower extremity edema, cardiac murmurs, jugular venous distension, or a laterally displaced/diffuse apical impulse. Electrocardiography may demonstrate abnormalities in heart rate, rhythm, or QRS morphology and duration, such as frequent atrial or ventricular premature beats, atrial fibrillation, or left ventricular hypertrophy. Chest radiography may show findings consistent with pulmonary congestion/edema or cardiomegaly. Echocardiography should confirm the presence of structural and/or functional cardiac abnormalities (3).

#### 2.4.3 Toxic-pathogenic syndrome

Diagnostic principles for the Toxic-pathogenic syndrome require a clinically confirmed diagnosis of heart failure with NYHA functional class II–IV. The pattern is established if the patient meets either of the following combinations: a. 1 major criterion + 2 minor criteria + 1 tongue/pulse criterion; OR b. 2 major criteria + 1 minor criterion + 1 tongue/pulse criterion (Table 2).

**Table 2:** Summary of diagnostic criteria for heart failure toxin syndrome.

Dimension	Criteria
Major symptoms	Agitation and restlessness; panting and shortness of breath; chest oppression as if blocked; cold limbs.
Minor symptoms	Orthopnea; lethargy; chest/flank fullness; facial or limb edema; dry mouth and bitter taste; grayish complexion; jugular venous distension; cyanosis of lips/nails; palpitations; fatigue; oliguria; dark-yellow urine; constipation; abnormal inflammatory findings (hs-CRP, IL-6, NLR, or inflammatory changes on chest imaging).
Tongue Appearance	Red tongue with yellow dry coating; dark or reddish-purple tongue; bluish-purple tongue; greasy coating; black coating; or tortuous/cyanotic sublingual veins.
Pulse	Knotted/intermittent, rapid, choppy, or racing pulse.

Assessment will be performed by qualified traditional Chinese medicine physicians at each center using the same scale. Assessors will receive centralized protocol and scoring training before recruitment, with refresher training and monitoring during the trial.

### 2.5 Inclusion criteria

1. Meets diagnostic criteria for hypertension and chronic heart failure (3, 10), with left ventricular ejection fraction (LVEF) ≤ 40% and serum NT-proBNP ≥450 pg/mL; Before randomization, participants must have clinically stable symptoms and comorbidities, patients with recent worsening heart failure or hospitalization may be screened only after clinical stabilization.
2. Meet the syndrome criteria for toxic-pathogenic syndrome in CM (https://www.cacm.org.cn/2024/12/02/30894/);
3. Age 18–75 years, no gender restrictions;
4. NYHA functional class II–III;
5. Voluntary participation with understanding and signed informed consent.

### 2.6 Exclusion criteria

1. Acute myocardial infarction (within 14 days);
2. Concurrent severe hepatic impairment (ALT > 100 U/L or AST > 100 U/L), severe renal impairment (glomerular filtration rate < 30 mL/min), or severe electrolyte imbalance (serum potassium > 5.5 mmol/L or < 3.5 mmol/L);
3. Severe hematologic disorders or malignancies;
4. Pregnancy or lactation;
5. Patients with psychiatric disorders;
6. Patients with severe heart failure and a life expectancy of less than six months;
7. Patients who have taken other Chinese herbal medicines or proprietary Chinese medicines with similar functions or components to the investigational drug within the past month;
8. Patients with known adverse reactions or allergies to the study drug;
9. Patients who have participated in other drug clinical studies within one month prior to enrollment.

### 2.7 Exit criteria

All subjects who have completed the informed consent form and been screened as eligible for the trial will not have their subsequent treatment affected regardless of when or why they withdraw. Patients have the right to withdraw from the study at any time and for any reason; however, unnecessary withdrawals should be minimized, and proactive measures should be taken to complete follow-up whenever possible to facilitate analysis of efficacy and safety. When a patient decides to withdraw, the investigator should contact the patient or their responsible relative via telephone or in-person visit to confirm the reason for withdrawal whenever possible. The investigator should collect any remaining medication upon withdrawal, complete the final assessment, finalize the case report, explain the reason for withdrawal, and follow up on any endpoint events occurring in the withdrawn patient. If the reason for withdrawal is an adverse event, it should be documented in the CRF.

### 2.8 Termination criteria

Treatment with the study drug may be discontinued under the following circumstances: (1) The patient may discontinue treatment at any time; (2) An allergic reaction clearly attributable to the study drug occurs; (3) Adverse symptoms or signs, or abnormal test results clearly attributable to the study drug occur, leading to a determination that discontinuation is necessary; (4) A woman of childbearing potential becomes pregnant during the study period.

The entire trial may be terminated at all sites if any of the following issues arise during the study: (1) The investigator identifies a serious safety concern; (2) A major protocol deviation occurs; (3) The sponsor discontinues funding or management support; (4) The regulatory authority withdraws approval for the trial.

### 2.9 Randomized design and blind method

This study employed stratified block randomization, with research centers as the stratification factor. The randomization table was generated by a statistician not involved in the study's statistical analysis using the PLAN procedure in SAS software. Samples were assigned according to group codes at a 1:1 ratio (Group A:Group B). Parameters such as block length and initial seed number, along with the randomization table itself, served as the primary blinding mechanism. The treatment assignments (experimental group, control group) corresponding to each group code constituted the secondary blinding mechanism. Both primary and secondary blinding layers were prepared in duplicate, sealed in opaque envelopes, and stored separately at the study coordinating institution and the sponsor. After screening and eligibility confirmation at each center, subjects were assigned random numbers sequentially based on enrollment timing and the center's allocated random number range. Medication corresponding to the assigned random number was then dispensed.

This study employs a double-blind design, meaning investigators, subjects, and all trial personnel remain blinded throughout the clinical trial. On-site drug blinding is guided by personnel from a statistical unit not involved in this study. The sponsor arranges for individuals unrelated to this trial to affix the pre-assigned drug codes onto labels. After packaging, each subject's medication will bear a unique drug code label corresponding to the drug code in the randomization table, which remains unchanged throughout the trial. Blinding records are maintained throughout the blinding process.

### 2.10 Treatment

After randomization, the treatment group received QSG, while the control group received a placebo version of QSG (2 sachets per dose, 5 g per sachet, twice daily, taken 30 min after breakfast and dinner respectively, dissolved in 150 mL of boiling water at 100 °C and stirred thoroughly). All participants received standardized treatment according to the Chinese Hypertension Prevention and Treatment Guidelines (2018 Revised Edition) (10)and the AHA/ACC/HFSA Heart Failure Management Guidelines 2022 (3). Unless contraindicated or not tolerated, the regimen will include the four foundational HFrEF drug classes: a renin–angiotensin system inhibitor, an evidence-based beta-blocker, a mineralocorticoid receptor antagonist, and an SGLT2 inhibitor. Antihypertensive treatment will follow the guidelines. Drug selection and doses will be individualized toward guideline-recommended target doses or the participant's maximum tolerated doses before enrollment. During the 12-week treatment period, investigators will aim to keep drug classes and doses stable. Any clinically necessary initiation, discontinuation or dose change, together with the date, reason and new dose, will be recorded in the CRF. Concomitant-medication exposure and major treatment changes will be summarized by group and evaluated in sensitivity analyses.

### 2.11 Storage and management of medications

Investigational medicinal products shall be stored under lock and key in a secure, controlled indoor area, protected from moisture. Each trial site must designate a responsible person for the management and storage of investigational medicinal products. Each time the investigator dispenses medication to a subject during a follow-up visit, the Medication Dispense Record must be completed, accurately and promptly recording the quantity dispensed and reminding the subject to return any remaining medication at their next visit. The quantity of returned medication shall be collected and verified, with the details recorded in the Medication Dispense/Return Record. At trial completion, all remaining medication must be returned to the sponsor with a completed Investigational Medicinal Product Recovery Form. Undistributed medication must be returned in its original sealed packaging. Remaining medication after trial completion shall be collected by the monitor for centralized disposal.

### 2.12 Outcome indicators

#### 2.12.1 Primary outcome

The main primary outcome is the NT-proBNP responder rate at week 12, defined as a decrease in NT-proBNP exceeding 30% from baseline. Collect fasting plasma from subjects and measure NT-proBNP levels following the reference kit protocol. The six-minute walk test assessed exercise tolerance by having patients walk as far as possible on a flat corridor within 6 min. Distances were categorized as: <150 m: Severe heart failure; 150–450 m: Moderate heart failure; >450 m: Mild heart failure.

#### 2.12.2 Secondary outcome

1. The following parameters were obtained via echocardiography: Left ventricular ejection fraction (LVEF), Left ventricular end-diastolic diameter (LVEDD), Left ventricular end-systolic diameter (LVESD), Interventricular septal thickness (IVS), Left ventricular posterior wall thickness (LVPW), Left ventricular short-axis fractional shortening (FS), Early diastolic peak mitral flow velocity/ late diastolic mitral flow peak (E/A), and early diastolic mitral flow velocity/tissue Doppler early diastolic annular velocity (E/e’).
2. NYHA Functional Class: The NYHA classification categorizes the impairment of cardiac function into four levels based on the level of activity that induces heart failure symptoms (12).
3. Kansas City Cardiomyopathy Quality of Life Questionnaire (KCCQ): KCCQ is a scoring tool used to assess the health status of patients with heart failure. It comprises 23 questions across seven domains: physical limitations, self-assessment, symptoms, self-awareness, psychological aspects, social functioning, and clinical assessment. Completion typically takes 4–6 min. Each question is scored out of 100 points, with higher scores indicating better quality of life.
4. Quantification Table for Toxic Pathogenic Factors in Heart Failure (https://www.cacm.org.cn/2024/12/02/30894/).

#### 2.12.3 Safety assessment

This trial will assess safety by monitoring adverse events (AEs), serious adverse events (SAEs), and discontinuation or treatment modifications due to AEs. Safety assessments at weeks 0, 4 and 12 will include vital signs, blood/urine/stool routine testing, 12-lead ECG, liver function, renal function, coagulation testing, and electrolytes including serum potassium and sodium; echocardiography and chest radiography are performed at weeks 0 and 12. All AEs will be graded from I (mild) to V (fatal), assessed for relationship to study treatment using the prespecified five-category causality scale, and followed until resolution, return to baseline or stabilization. SAEs will be reported to the monitor, coordinating center and manufacturer within 24 h. Independent monitors and auditors will oversee protocol adherence, source-data verification and safety reporting; this oversight is used instead of a separate data safety monitoring committee.

#### 2.12.4 Long-term follow-up outcomes and event adjudication

At weeks 26 and 52, investigators will collect the number and dates of heart-failure rehospitalizations and major cardiovascular events through scheduled contact, participant/family interview and review of hospital source records. Heart-failure rehospitalization is defined as an unscheduled admission for a primary diagnosis of worsening heart failure lasting >24 h, supported by new/worsening symptoms and signs and intensification of heart-failure therapy. All-cause rehospitalization is any unscheduled inpatient admission meeting the same duration rule, irrespective of cause. Cardiovascular events include cardiovascular death, nonfatal myocardial infarction, nonfatal stroke, resuscitated cardiac arrest, malignant arrhythmia and heart-failure rehospitalization. A blinded clinical endpoint committee, composed of three cardiologists blinded to treatment allocation, will adjudicate events from source documents. Disagreements will be resolved by consensus or by a fourth senior adjudicator. Only committee-adjudicated events will be used in the primary analyses of rehospitalization and cardiovascular outcomes.

### 2.13 Data management

During each study visit, patient demographics and all clinical data will be recorded and maintained in physical medical record folders. Data will then be entered into Epidata software to centralize all study data. The data management process will adhere to regulatory requirements outlined in the Good Clinical Practice (GCP) guidelines and the Technical Guidelines for Clinical Trial Data Management, ensuring the authenticity, completeness, accuracy, and traceability of data.

### 2.14 Quality control in research

Investigators shall fulfill their respective responsibilities and strictly adhere to the clinical research protocol, employing standard operating procedures. All relevant observations and findings must be verified to ensure the implementation of the clinical research quality control and quality assurance system. Investigators must provide necessary training to all personnel involved in the clinical research, explaining relevant materials, operational standards, and responsibilities to ensure data is recorded truthfully, accurately, completely, promptly, and lawfully in medical records and CRFs. Subject allocation in clinical research must follow the randomized allocation plan specified in the study design. The treatment group coding for each subject should be maintained as a blinded baseline by both the statistical unit and the investigator. Monitors shall follow standard operating procedures to oversee protocol compliance, confirm the accuracy and completeness of all data recording and reporting, and ensure CRFs are correctly completed and consistent with source documents. Auditors shall conduct systematic reviews of clinical research activities and documentation to evaluate compliance with the protocol, SOPs, and relevant regulatory requirements. All laboratory test data in clinical research must be accurate and either documented in the case report form or supported by original report copies attached thereto. Medical statisticians shall incorporate study data into reports completely and without error. All steps involving data management must be documented to facilitate verification of data quality and implementation processes.

### 2.15 Data analysis and statistical methods

SPSS 24.0 software will be used to perform statistical analysis on the collected data. All statistical tests will be two-tailed, with a *P*-value ≤ 0.05 indicating statistical significance for the tested difference. Descriptive analysis: Categorical indicators will describe the number and percentage of cases in each category. Quantitative indicators will be described using mean, standard deviation, maximum value, minimum value, median, lower quartile (Q1), and upper quartile (Q3). Comparisons between two groups of general characteristics will be analyzed using appropriate methods based on the type of indicator. For quantitative data, between-group comparisons will employ paired t-tests or Wilcoxon signed-rank tests. For categorical data, chi-square tests or exact probability methods will be used. For ordinal data, Wilcoxon signed-rank tests or the Mann–Whitney U (MWU) test will be applied. Age, atrial fibrillation status and estimated glomerular filtration rate will be recorded at baseline and examined in covariate-adjusted and sensitivity analyses because these factors can materially alter NT-proBNP concentrations. Time to first event will be displayed with Kaplan–Meier curves, compared by the log-rank test, and modeled using Cox regression to estimate hazard ratios (HRs) with 95% confidence intervals. Event counts and recurrent rehospitalizations will be summarized descriptively by group.

The Full Analysis Set (FAS) followed the intention-to-treat principle, including all randomized subjects who received at least one dose of the study drug, with exclusions limited to major protocol violations, no study drug exposure, or complete lack of post-randomization data. The per-protocol set (participants with ≥80% adherence, primary endpoint data and no major protocol violation) will support sensitivity analyses, and the safety set will include all randomized participants who received at least one dose and had a safety assessment. For missing co-primary endpoint data, the prespecified conservative worst-case approach will be used in the primary FAS analysis. Missing data amount, reasons and patterns will be reported by group. Multiple imputation under a missing-at-random assumption will be used as a sensitivity analysis, with additional best/worst-case analyses to evaluate departures from that assumption.

## 3 Discussion

HFrEF represents a severe manifestation or end-stage condition of various cardiac diseases (13). Long-term hypertension predisposes to pathological changes such as ventricular remodeling, leading to heart failure with reduced ejection fraction (14). Although ACEIs/ARBs, beta-blockers, and SGLT2 inhibitors have become cornerstones of standard HFrEF therapy, long-term patient outcomes remain suboptimal with persistently high readmission rates (15, 16). CM demonstrates favorable effects on long-term efficacy and readmission rates in heart failure, though individual responses vary considerably.

Based on CM theory, we integrate the primary symptom clusters of HErEF—restlessness, dyspnea, chest tightness, cold extremities, orthopnea, lethargy, edema, dry mouth with bitter taste, sallow complexion, jugular vein distension, cyanosis of lips and nails, palpitations, fatigue, oliguria, elevated high-sensitivity C-reactive protein (CRP), elevated interleukin (IL)-6, and elevated neutrophil-to-lymphocyte ratio (NLR) in patients with hypertension and HErEF. We propose a “toxic” state in these patients and developed the compound formula QSG with “Yiqi Jiedu” functions for heart failure treatment. Preliminary studies confirmed the efficacy and safety of QSG, but high-quality clinical evidence regarding its therapeutic effect in hypertension associated HFrEF remains lacking. Therefore, with support from the Ministry of Science and Technology, we conducted this multicenter, double-blind, randomized placebo-controlled trial to investigate the efficacy and safety of QSG in hypertension associated HFrEF patients.

The primary characteristic of HFrEF is reduced exercise tolerance resulting from impaired cardiac and vascular function. Dyspnea and fatigue are the main manifestations of exercise intolerance and also hallmark symptoms of HFrEF. This study employs the six-minute walk test as one of the primary outcome measures to objectively assess exercise tolerance in hypertension associated HFrEF patients. Additionally, we selected the change in NT-proBNP from baseline to the end of treatment as a key outcome indicator. As the most valuable and reliable laboratory biomarker for heart failure recommended by guidelines, NT-proBNP is characterized by ease of measurement, accuracy, and reproducibility (17, 18). It is commonly used to diagnose heart failure, stratify risk, and evaluate the clinical efficacy of medications.

NYHA cardiac function classification serves as a widely accepted indicator for assessing impaired cardiac function (19, 20). Echocardiography accurately reflects ventricular remodeling and cardiac function status (21), making it one of our secondary outcome measures. Additionally, this study comprehensively evaluated patients’ living conditions, social quality, and physical changes. Therefore, the KCCQ and the Heart Failure Toxic Syndrome Quantification Scale were also considered as secondary outcome measures.

This study involves multiple participating institutions, making quality control one of our primary challenges. We established Standard Operating Procedures (SOP) and provided extensive training to all investigators/physicians prior to patient recruitment. Through initial, supplemental training, and ongoing review, we ensured proper execution of SOP to guarantee data collection quality. Furthermore, we prioritized patient communication to ensure all participants had a thorough understanding of the trial, enabling strict adherence to requirements and attendance at all scheduled appointments.

This study design strictly adheres to the CONSORT guidelines employing a multicenter, randomized, double-blind, placebo-controlled trial design to minimize bias (22). The sample size calculation is scientifically sound, with a follow-up period extending to 52 weeks, enabling thorough assessment of long-term efficacy and safety outcomes. Furthermore, we incorporated readmission rates as a late-stage follow-up indicator, directly linking to health economics metrics, thereby demonstrating strong clinical and practical significance. We anticipate this clinical trial will provide high-level evidence-based medical evidence for the application of traditional Chinese medicine in the field of hypertension-related heart failure.
